# Retraction

**Published:** 2011

**Authors:** Tehemton E. Udwadia

**Affiliations:** Editor-in-Chief, Journal of Minimal Access Surgery

## Urgent Communication From Editor-in Chief Regarding Duplicate Publication

On behalf of the Editorial Team of the Journal of Minimal Access Surgery (JMAS) we regret to announce that the following paper published in the October - December 2009 issue of the Journal of Minimal Access Surgery (JMAS) is hereby retracted.

## Deepak J, Agarwal P, Bagdi RK, Balagopal S, Madhu R, Balamourougane P. Pediatric cholelithiasis and laparoscopic management: A review of twenty two cases. J Minim Access Surg. 2009;5:93-6.

In January 2011 we were informed by the Editor of Journal of Indian Association of Pediatric Surgeons (JIAPS) that following paper carrying very similar material from the same authors was published in that journal.

## Gowda DJ, Agarwal P, Bagdi R, Subramanian B, Kumar M, Ramasundaram M, Paramasamy B, Khanday ZS. Laparoscopic cholecystectomy for cholelithiasis in children. J Indian Assoc Pediatr Surg. 2009;14:204-6.

As both the papers are almost identical, with minor differences in terms of the number of patients treated, this appears to be a clear case of duplicate publication. In fact, upon tracing the timeline of submission of the papers in JMAS and JIAPS it became apparent that they were submitted to the journals on consecutive days.

We communicated with the first author of the article published in the JMAS seeking an explaination. As none was forthcoming even after six weeks, it was decided to retract the article from JMAS. The Editor of the JIAPS has been apprised of our decision and the Head of the Institution where the publication originated has been informed of the professional misdemeanor on part of their Faculty members. As is customary in such cases, the indexing authoroties, including PubMed have been intimated of the retraction of the paper from JMAS.

We sincerely regret the time peer reviewers and others spent evaluating this paper and hope that there are no instances of duplicate publication in the JMAS in the future.

